# Clinical Correlation and Metabolic Findings on 18F-Fluorodeoxyglucose Brain PET in Dementia With Lewy Bodies: A Case Report

**DOI:** 10.7759/cureus.89795

**Published:** 2025-08-11

**Authors:** David Gutierrez Albenda, Paula Ulate Blanco, Julyana Murillo, Juan J Solano Brenes, Nelson Mauricio Sánchez Hidalgo

**Affiliations:** 1 Cyclotron PET/CT Laboratory, University of Costa Rica, San José, CRI; 2 School of Medicine, Faculty of Medicine, University of Costa Rica, San José, CRI; 3 Faculty of Medicine, University of Costa Rica, San José, CRI; 4 School of Medicine, University of Costa Rica, San José, CRI

**Keywords:** ¹⁸f-fdg pet, cerebral hypometabolism, cingulate island sign, dementia with lewy bodies, differential diagnosis, functional neuroimaging, neurodegenerative disorders

## Abstract

Neurodegenerative dementias comprise a heterogeneous group of diseases with distinct pathological substrates, among which Alzheimer’s disease, dementia with Lewy bodies (DLB), and frontotemporal dementia are the most prominent. The early and accurate diagnosis of these conditions remains a clinical challenge, in which both structural and functional imaging techniques play crucial roles. This article presents a case of a 68-year-old female patient with progressive cognitive decline, evaluated using ¹⁸F-fluorodeoxyglucose positron emission tomography (¹⁸F-FDG PET), which revealed a characteristic pattern of symmetric hypometabolism in the temporal, parietal, and occipital lobes, with preserved metabolism in the posterior cingulate cortex (the cingulate island sign), a finding highly suggestive of DLB. This case highlights the value of ¹⁸F-FDG PET as a diagnostic tool in the differential evaluation of dementias and underscores the importance of recognizing the specific metabolic pattern of DLB to support timely clinical decision-making. The model incorporating visual and semi-quantitative parameters achieved a diagnostic accuracy of 84.3%.

## Introduction

Main neurodegenerative pathologies associated with the most common dementia syndromes include beta-amyloid accumulation and paired helical filament tau (PHF-tau) neurofibrillary tangles in Alzheimer’s disease (AD); Lewy bodies in dementia with Lewy bodies (DLB) and in Parkinson’s disease dementia (PDD); and TDP-43 deposits in the neuropathological changes related to limbic-predominant age-related TDP-43 encephalopathy (LATE) [1].

On the other hand, frontotemporal dementia (FTD), although less prevalent, presents a range of complex pathological alterations, with frontotemporal lobar degeneration (FTLD)-tau and FTLD-TDP being the most frequent. In contrast, cognitive impairment of vascular origin and vascular dementia (VaD) are primarily attributed to ischemic lesions such as cerebral infarcts, although hypoxic and hemorrhagic damage may also be involved [1].

A comprehensive assessment is recommended to achieve an accurate diagnosis, including the collection of collateral history and complementary studies such as neuroimaging, lumbar puncture, neuropsychological evaluation, and genetic testing when indicated [2]. Notably, in addition to cognitive impairment, approximately 90% of patients with dementia present with neuropsychiatric or behavioral symptoms, such as psychosis, aggression, agitation, and depression [3].

Early and accurate diagnosis of the specific dementia subtype is essential to optimize clinical care and facilitate the development of more effective therapies. In this context, structural and molecular imaging techniques have significantly contributed to a deeper understanding of the pathophysiology of neurodegenerative dementias, and their use in clinical practice has become increasingly common as a diagnostic tool in the early stages of the disease [4].

Amyloid positron emission tomography (PET) is typically positive in AD and in some cases of DLB, but generally negative in FTD. Tau PET tracers show stronger signals in AD and weaker signals in FTD. Volumetric loss quantification using magnetic resonance imaging (MRI) and the assessment of cerebral metabolism through ¹⁸F-fluorodeoxyglucose positron emission tomography (¹⁸F-FDG PET) help differentiate among various causes of dementia and monitor disease progression. Additionally, neuroinflammation can be evaluated using PET [5].

¹⁸F-FDG PET has demonstrated greater sensitivity and specificity compared to structural MRI, particularly when both modalities are used complementarily to enhance diagnostic performance. In the context of FTD, PET offers superior sensitivity compared to single-photon emission computed tomography (SPECT) and is primarily used as an adjunct to clinical and other imaging assessments [6]. Dementia remains one of the leading causes of disability and mortality worldwide [4]. This article highlights the importance of imaging techniques in the diagnostic workup of dementia.

## Case presentation

A 68-year-old female patient with a history of cognitive decline spanning more than 10 years underwent evaluation at our center. She reported progressive difficulties in attention, concentration, and recent memory. Her past medical history also included a depressive disorder of over 15 years, under ongoing medical and pharmacological management. Previous imaging studies had not revealed significant findings.

On April 1, 2023, the patient was referred for brain imaging to aid in the differential diagnosis of a possible neurodegenerative disease. The patient had complied adequately with the pre-scan preparation protocol, followed all instructions, and did not receive any medication before administration of the radiotracer ¹⁸F-fluorodeoxyglucose (¹⁸F-FDG).

A high-resolution PET scan was subsequently performed using LySO crystal detectors, with axial acquisition at a speed of 1 mm/s. PET reconstruction was carried out using the TrueX+TOF (ultraHD-PET) algorithm, with two iterations, five subsets, zoom 1, a 5.0 mm Gaussian filter, and attenuation correction. The PET scan was complemented by a low-dose CT scan using a 128-slice helical multidetector, with 5 mm axial slices and a pitch of 0.6 mm. CT image reconstruction was performed using 2 mm slices with the SAFIRE (Sinogram Affirmed Iterative Reconstruction) iterative method, Body Regular (BR34) reconstruction filter, mediastinal window, 500 mm field of view (FOV), 1 mm increment, and intensity level 3.

**Figure 1:**
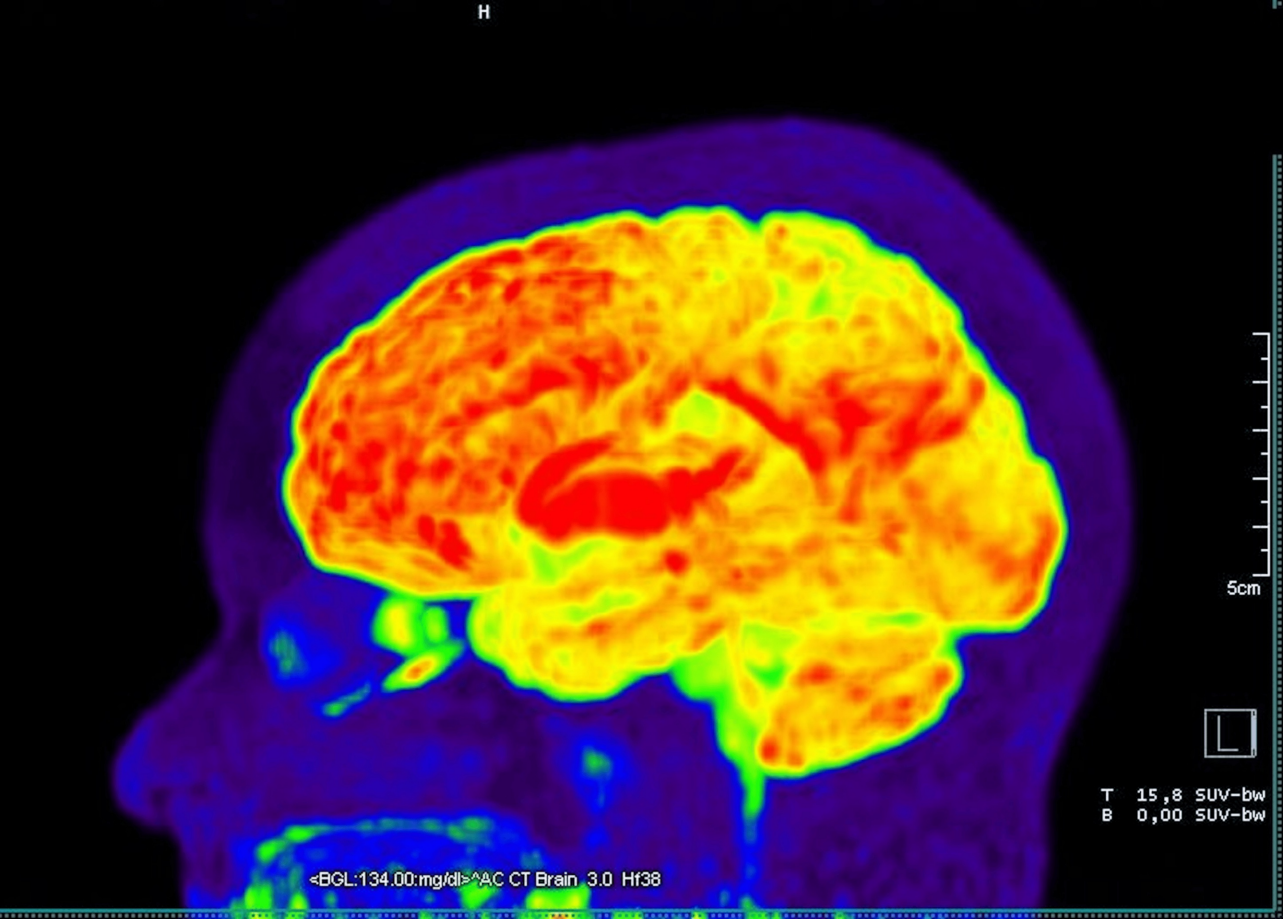
Lateral view maximum intensity projection (MIP) image of ¹⁸F-fluorodeoxyglucose brain PET showing cortical hypometabolism in the temporal lobes and association cortices, extending symmetrically and bilaterally to the superior parietal and occipital regions.

**Figure 2:**
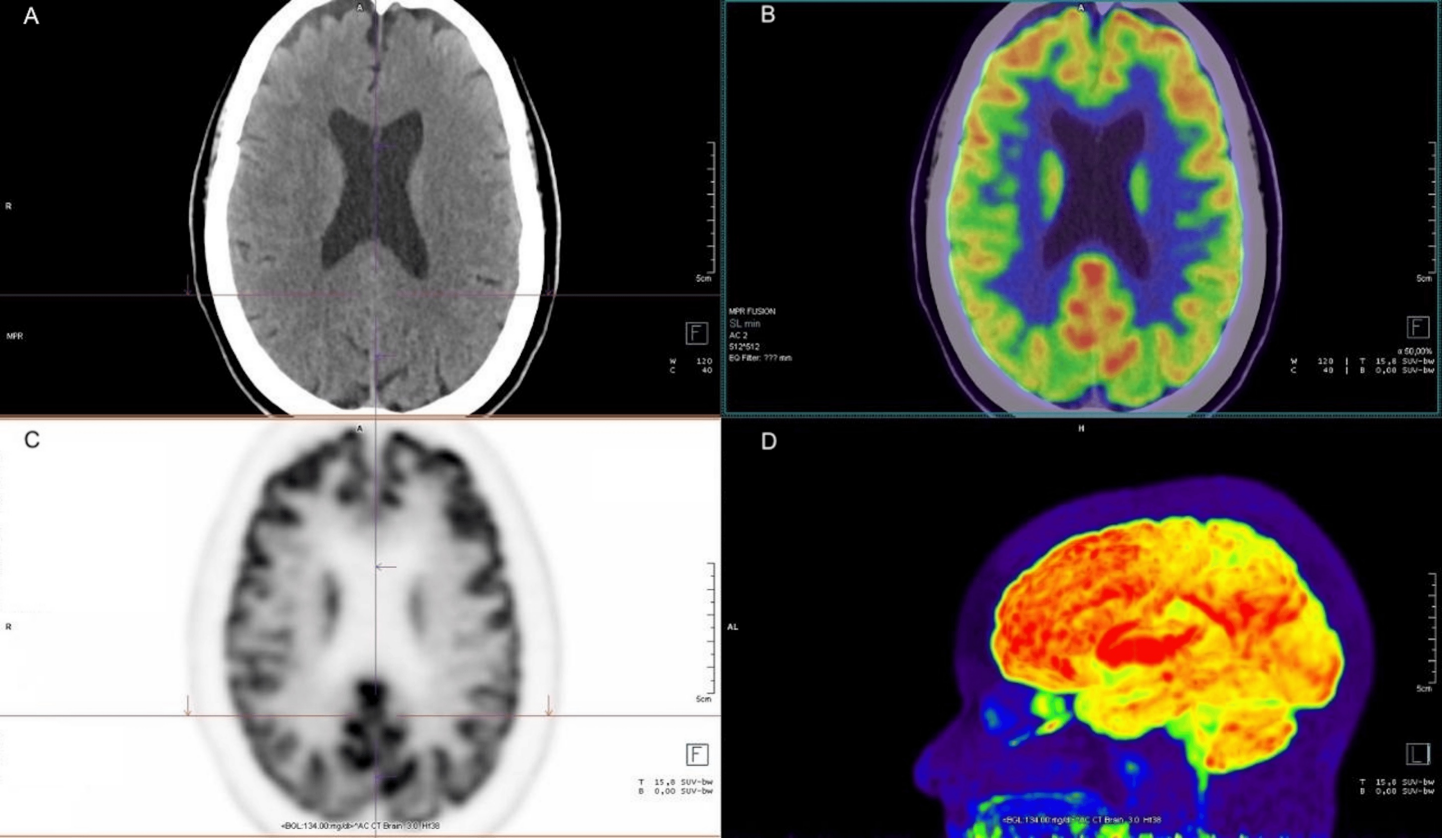
(A) Axial cranial CT scan showing no significant structural abnormalities. (B) Axial ¹⁸F-fluorodeoxyglucose (¹⁸F-FDG) brain PET image showing the cingulate island sign, with crosshairs marking the area of preserved cingulate metabolism. (C) Fused ¹⁸F-FDG PET/CT brain images. (D) Lateral view maximum intensity projection (MIP) image of the ¹⁸F-FDG brain PET study.

The ¹⁸F-FDG PET scan revealed marked bilateral hypometabolism in the cerebral cortex of both temporal lobes, with a symmetric pattern and moderate to severe intensity. This alteration extended to the association areas and reached the superior parietal regions bilaterally, also symmetrical and of moderate intensity. Additionally, diffuse moderate bilateral hypometabolism was observed in the occipital cortex (Figure 1). The remaining cortical regions of the cerebral hemispheres, as well as the cerebellum, basal ganglia, thalami, and anterior and posterior cingulate areas (cingulate island sign), exhibited preserved radiotracer uptake, with no significant metabolic abnormalities (Figure 2). It is worth noting that the observed hypometabolic changes were more pronounced than the atrophic findings identified in structural imaging studies.

The ¹⁸F-FDG PET scan was conducted at a specialized referral center with access to limited clinical information. Details regarding the patient’s pharmacological regimen, therapeutic response, and clinical follow-up were not provided by the referring physician and were therefore unavailable at the time of imaging.

## Discussion

The metabolic pattern observed on the ¹⁸F-FDG PET/CT in this case is highly suggestive of DLB, characterized by posterior cortical hypometabolism with relative preservation of the posterior cingulate cortex, a finding known as the “cingulate island sign” (CIS). This feature is particularly relevant because it helps differentiate DLB from other dementias, especially AD, which typically presents with marked hypometabolism in the posterior cingulate cortex. The recognition of this distinctive metabolic pattern, even in cases with atypical clinical presentation, can significantly enhance diagnostic confidence and support early therapeutic decision-making.

DLB is the second most common cause of degenerative dementia, after AD [7]. It is characterized by the intracellular accumulation of Lewy bodies, inclusions primarily composed of phosphorylated alpha-synuclein, which interfere with neuronal function and cause cognitive, motor, and neuropsychiatric manifestations that significantly impact quality of life [8]. Although initially these inclusions were associated with idiopathic Parkinson’s disease, their cortical distribution and close relationship with cognitive decline are now recognized, supporting their role in syndromes such as DLB and PDD [1].

Molecular imaging techniques, such as SPECT and ¹⁸F-FDG PET, have been fundamental in understanding DLB pathophysiology by allowing real-time assessment of cerebral metabolic activity with specific radiotracers [9]. Metabolic studies in this dementia have shown a distinctive pattern of hypometabolism predominantly affecting the occipital cortex, visual association areas, and the posterior parietotemporal region [9,10]. Among the most useful neuroimaging findings for differentiating DLB is the CIS, characterized by relative preservation of metabolism in the posterior cingulate cortex, contrasted with hypometabolism in adjacent regions. This pattern creates the appearance of a metabolically active “island” and is a highly specific marker for distinguishing DLB from other dementias, particularly AD [9,11]. Although there is no standardized method for its quantification, the CIS ratio is traditionally calculated as the relationship between metabolism or perfusion in the posterior cingulate cortex (PCC) and the sum of metabolism or perfusion in the precuneus and cuneus (PpC). PET ¹⁸F-FDG studies have reported this metric’s diagnostic accuracy ranging from 72% to 78% for differentiating DLB from AD [12]. In this case, preserved metabolism in the posterior cingulate cortex aligns with this characteristic pattern, further reinforcing the diagnostic suspicion.

A recent study by Mattoli et al. (2023) evaluated the diagnostic performance of FDG-PET in differentiating AD and DLB. They analyzed 61 patients (34 with DLB and 27 with AD) and found that visual DLB signs on FDG-PET were significantly more frequent in DLB patients, achieving a diagnostic accuracy of 86.9%. Semi-quantitative analysis revealed reduced uptake in key regions, including the right precuneus and visual areas. The model based on visual and semi-quantitative features showed a diagnostic accuracy of 84.3% [13].

These findings not only strengthen the differential diagnosis between DLB and other dementias but also highlight the importance of functional neuroimaging techniques, particularly FDG-PET, as key tools for early and accurate clinical evaluation of dementia syndromes.

## Conclusions

Clinically, DLB and AD present with overlapping features; however, their management strategies differ significantly, underscoring the importance of an accurate differential diagnosis. This case highlights the clinical utility of ¹⁸F-FDG PET as a diagnostic tool in the differential evaluation of neurodegenerative dementias. Although the clinical manifestations were not strongly suggestive of DLB, the characteristic hypometabolic pattern observed on PET imaging, affecting the temporal, parietal, and occipital cortices, with preserved metabolism in the posterior cingulate cortex (cingulate island sign), provided key diagnostic support for this condition. These findings, consistent with clinical data and current literature, reaffirm the relevance of functional imaging techniques in the early and accurate characterization of dementia syndromes, which is essential to guide individualized therapeutic strategies and improve patient prognosis.
